# Prognostic Assessment with the Malnutrition Universal Screening Tool in Heart Transplant Recipients: A Pilot Study and a Single-Center Experience

**DOI:** 10.3390/jpm14121140

**Published:** 2024-12-05

**Authors:** Assunta Fabozzo, Valentina Lombardi, Giorgia Cibin, Emma Bergonzoni, Giulia Lorenzoni, Dario Gregori, Chiara Tessari, Daniela Bacich, Augusto D’Onofrio, Giuseppe Toscano, Antonio Gambino, Vincenzo Tarzia, Nicola Pradegan, Gino Gerosa

**Affiliations:** 1 Cardiac Surgery Unit, MCS and Heart Transplant Program, Padova University Hospital, 35128 Padova, Italy; assunta.fabozzo@aopd.veneto.it (A.F.); giorgia.cibin@aopd.veneto.it (G.C.); emma.bergonzoni@studenti.unipd.it (E.B.); chiara.tessari@unipd.it (C.T.); antonio.gambino@unipd.it (A.G.); gino.gerosa@unipd.it (G.G.); 2 Unit of Biostatistics, Epidemiology and Public Health, Department of Cardiac, Thoracic, Vascular Sciences and Public Health, University of Padova, 35128 Padova, Italy; giulia.lorenzoni@unipd.it (G.L.); dario.gregori@unipd.it (D.G.)

**Keywords:** malnutrition, nutritional screening, cardiac surgery, heart transplant

## Abstract

**Introduction and aims**: Malnutrition is associated with increased morbidity and mortality in patients who undergo cardiac surgery. Nevertheless, objective assessment of malnourished patients undergoing heart transplantation (HT) is limited. We aimed to analyze the relationship between the malnutrition status and the early and late clinical outcomes of patients undergoing HT using a novel semi-quantitative tool. **Methods**: All patients aged ≥18 years who underwent HT between January 2015 and July 2020 in a single center were retrospectively evaluated and included in the study. The semi-quantitative Malnutrition Universal Screening Tool (MUST) score (already validated in heart failure) was calculated for each patient at the time of transplantation to assess their nutritional status. A propensity score weighting approach was performed to evaluate the association between the increase in MUST score and the risk of early complications and in-hospital mortality. A Cox regression analysis was performed to assess follow-up mortality. **Results**: A total of 168 HT patients (median age 58.4 years, IQR 49.5–65.2, men *n* = 128, 76%) were included within the study period. Their median preoperative BMI was 24.0 kg/m^2^ (IQR 21.2–27.9). Preoperative MUST scores of 0, 1, and ≥2 were found in 92 (55%), 24 (14%), and 52 (31%) patients, respectively. The median preoperative eGFR was 64.3 mL/min (IQR 49.0–83.2). An increase in MUST score (from 0 to 2) was not significantly related to major postoperative complications or in-hospital mortality. An analogous increase in MUST score was associated with increased follow-up mortality risk (hazard ratio 1.28, 95% CI 1.04–1.83, *p* = 0.024). **Conclusions**: Malnutrition assessed with the MUST score seems not to be associated with increased in-hospital mortality or major postoperative complications in patients who undergo HT, but according to our preliminary data it is related to patients’ long-term mortality.

## 1. Introduction

**Figure 1:**
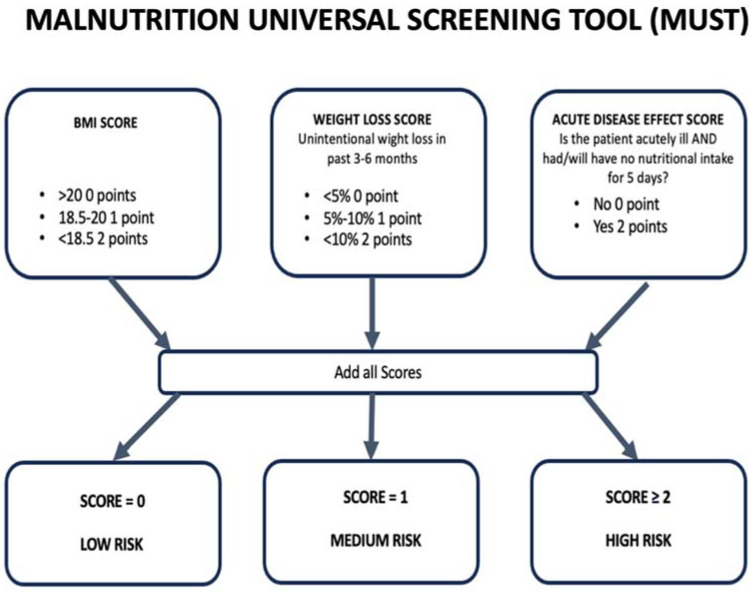
Malnutrition Universal Screening Tool (MUST) variables and score.

Malnutrition is a common comorbidity among patients with end-stage heart failure (HF), for whom the best therapeutic option remains cardiac transplantation (HT) [1,2]. Historically, the nutritional status in this patient population has been assessed using the body mass index (BMI) [3], hypoalbuminemia [4], or anemia [5]. However, as highlighted by other authors [6], none of these parameters alone serves as a reliable indicator of nutritional status in HF patients, as all can be significantly altered as a consequence of the disease itself. Serum albumin concentration may be influenced by intrinsic conditions of heart failure, such as chronic inflammation, fluid overload, and hepatic congestion, while variations in blood volume can affect BMI. Despite specific mechanisms underlying malnutrition in HF patients [6], this condition still affects 10–25% of patients undergoing surgery and 25–60% of patients with HF [7,8,9,10,11,12], resulting in a higher risk of postoperative infections, non-infectious complications, increased mortality rates, and poorer quality of life. Therefore, it is essential to identify malnourished patients before HT surgery to maximize the benefits of transplantation [13]. Among the various indices clinically available to assess nutritional status in patients [14], the Malnutrition Universal Screening Tool (MUST) appears to be the most dynamic and suitable tool for our purpose, as it considers three main aspects comprehensively: (i) body mass index (BMI), (ii) unintentional weight loss (UWL), and (iii) the expectation of no nutritional intake for at least 5 days in case of acute illness (Figure 1). However, this score has never been applied to heart transplant (HT) candidates and recipients. Therefore, we aimed to assess the relationship between the malnutrition status before HT, established using the MUST score, and early and late post-transplant outcomes.

## 2. Methods

### 2.1. Study Design, Population, and Data Collection

We performed a retrospective clinical study on all adult patients (≥18 years) who underwent HT between January 2015 and July 2020 at our department. The analysis included all patients for whom the MUST score could be calculated before the HT. Pre-transplant, intraoperative, and postoperative clinical data were extracted from a local database and supplemented by a review of medical records. Follow-up clinical data were obtained directly during ordinary follow-up visits or by telephone. The authors did not receive funding for this study and had no financial interests. This study was approved by our local ethical committee.

### 2.2. Malnutrition Universal Screening Tool (MUST)

The MUST score was used to assess patients’ nutritional status before HT. This tool was developed by the British Association for Parenteral and Enteral Nutrition (BAPEN), and it classifies patients into malnutrition risk levels based on BMI, the existence of a history of involuntary weight loss, and the likelihood of future weight loss secondary to acute illness, conditional on the absence of food intake for more than 5 days. Each item is scored from 0 to 2 points as follows: body mass index (BMI) > 20 kg/m^2^ = 0, 18.5–20 kg/m^2^ = 1, <18.5 kg/m^2^ = 2; weight loss <5% = 0, 5–10% = 1, >10% = 2; acute illness and its relation to food intake in the following five days: absence = 0, presence = 2. Low-risk patients are classified as 0 points, medium-risk patients = 1 point, and high-risk patients = ≥2 points, as explained in Figure 1.

### 2.3. Statistical Analysis

Descriptive statistics were reported as I quartile/median/III quartile for continuous variables, and as absolute numbers (percentages) for categorical variables. To account for potential confounding factors, a propensity score weighting approach was used. Propensity scores were estimated using the covariate balancing propensity score (CBPS) [15], and a trimming of the weights was performed at the 90th quantile. Variables considered for propensity score estimation are reported in Table 1. Covariate balance was evaluated using treatment–covariate correlation.

A weighted logistic regression approach was used to evaluate the effects of the MUST score on binary outcomes. Results were reported as odds ratios (ORs), 95% confidence intervals (CIs), and *p*-values. Weighted gamma models were employed to assess the effects of the MUST score on continuous outcomes, since their distribution was found to be non-normal. The marginal effect was computed by considering the partial derivatives of the marginal expectation. Results were reported as average marginal effects (AMEs), 95% CIs, and *p*-values. Analyses were performed using R software [16] utilizing the packages rms, CBPS, and WeightIt [17] for propensity score weighting procedure estimation, and margins [18] for AME computation. A Kaplan–Meier survival curve was generated to evaluate the survival rates at 1, 3, and 5 years, and a Cox regression analysis was conducted to assess follow-up mortality.

## 3. Results

During the study period, 168 patients were included. Demographic data and preoperative characteristics are reported in Table 2. The median age of the entire patient cohort was 58.4 years, (IQR 49.5–65.2). The majority were men (n = 128, 76%). According to the preoperative MUST score, 92 patients (55%) were at low risk of malnutrition (MUST score = 0), 24 patients (14%) were at moderate risk (MUST score = 1), and 52 patients (31%) were at high risk (MUST score ≥ 2). The median serum albumin and creatinine levels were 34.0 g/L (28.0–38.8) and 103.5 mg/dL (87.0–127.3), respectively. Seventeen patients (10%) required ECMO implantation before HT for cardiogenic shock, 23 patients (14%) required a paracorporeal left ventricular assist device (LVAD) before HT, and 50 patients (30%) required an intracorporeal LVAD. Additionally, 57 patients (34%) of the entire cohort were already hospitalized before HT, with a median of 17 days (8.5–36.5) of pre-transplant hospitalization.

The donors’ median age was 52.0 years (IQR 39.8–60.3). The median ischemia time of the organ was 220.0 min (IQR 176.0–246.0). The median durations of cardiopulmonary bypass and aortic cross-clamp were 198.5 min and 80 min, respectively (Table 1).

The postoperative characteristics reported in Table 3 show that 40 patients (24%) required ECMO within the first 24 h after HT, and an additional 6 patients (4%) needed this procedure beyond the first 24 h. The median ICU stay was 5.0 days (IQR 3.0–16.0), with a median of 2 days (IQR 1.0–4.0) of mechanical ventilation. Forty-seven patients had positive blood cultures, with five (3%) occurring within the first 24 h and forty-two (25%) occurring afterward. Among these, 17 patients (36%) were found to have multidrug-resistant (MDR) bacteria. Additionally, 12 patients (7%) developed postoperative pneumonia. A total of 56 patients (33%) required dialysis treatment via CVVH, with 25 patients (15%) needing it within the first 24 h post-transplant, and 31 patients (18%) afterward. The median hospital stay was 35.0 days (IQR 28.0–49.3). The in-hospital mortality rate was 25 patients (15%).

**Figure 2:**
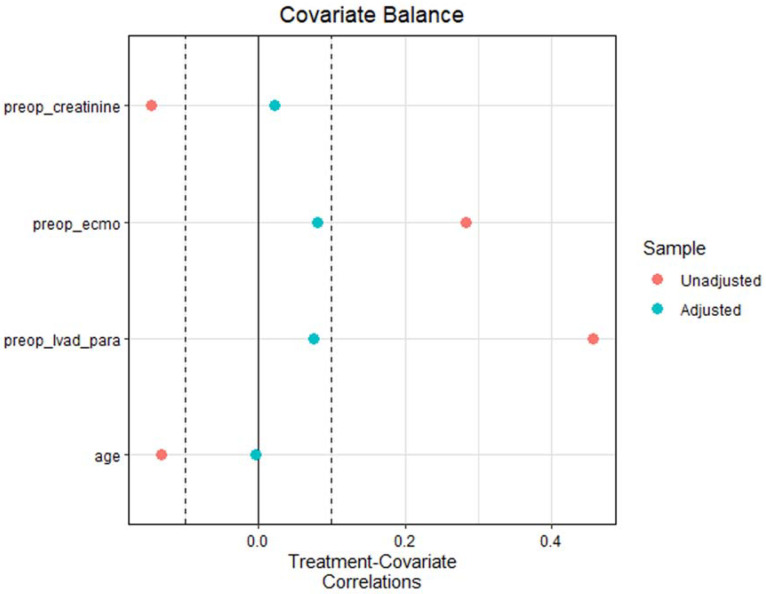
Covariate balance after the propensity score weighting procedure.

The propensity score weighting procedure resulted in a good balance of the covariates (Figure 2).

Interestingly, the weighted logistic regression analysis did not show any postoperative complications significantly associated with the MUST score. Furthermore, in-hospital postoperative mortality was not significantly associated with the MUST score (Table 3).

**Figure 3:**
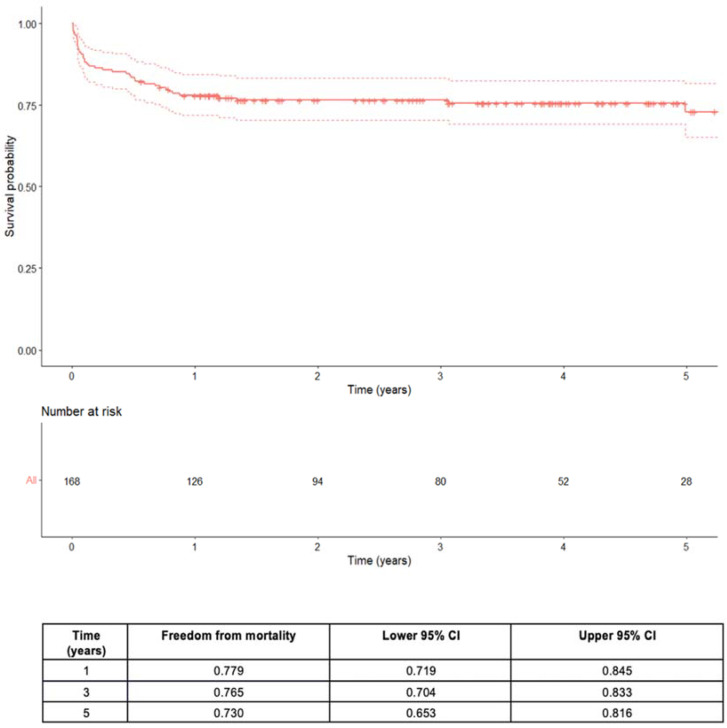
Kaplan–Meier overall follow-up survival curves in patients after heart transplant (legend: CI = confidence interval).

At a median follow-up time of 3 years (0–7 years), the 3- and 5-year survival rates were 77% (95% CI 70–83, patients at risk *n* = 80) and 73% (95% CI 65–81, patients at risk *n* = 28), respectively (Figure 3). Finally, regarding the mortality risk at follow-up, we found a statistically significant association through Cox regression analysis (hazard ratio 1.38, 95% CI 1.04–1.83, *p* = 0.024) (Table 4) with the increasing MUST value measured throughout the follow-up period.

## 4. Discussion

Malnutrition is a complex clinical condition that can significantly affect the outcomes of patients undergoing cardiac surgery. The MUST is a useful and sensitive instrument for identifying and assessing the risk of malnutrition [19]. In our pilot study, we aimed to determine whether it could also be used for patients undergoing HT. We included 168 heart transplant patients from 2015 to 2020 and found that 45% of them had a MUST score of 1 or higher. Our analysis did not reveal a direct link between the MUST score and major in-hospital complications, including mortality. However, our pilot study did show that a higher MUST score was associated with an increased risk of mortality during follow-up.

The potential predictive value of the MUST score in surgical patients has already been demonstrated by several authors who found a correlation between a low BMI and long-term mortality in patients undergoing cardiac valve surgery [9], as well as a correlation between low serum albumin levels and in-hospital mortality in patients undergoing coronary artery bypass grafting [20]. However, few studies have examined the specific subpopulation of patients with end-stage HF undergoing HT. The assessment of malnutrition status in this category of patients can be conducted using indices such as serum albumin or BMI.

In this regard, Kato et al. [21] found that serum albumin levels below 35 g/L were associated with decreased one-year survival after HT. However, serum albumin and BMI are static indices, and relying solely on these two parameters in this patient population is limited, as they are strongly influenced by fluid shifts and systemic inflammation inherent to end-stage HF. The MUST score, by evaluating various key factors such as body mass index (BMI), unintentional weight loss, and the presence of acute conditions that can affect food intake, is considered to be a simple, easy-to-apply tool with high prognostic value for identifying malnourished patients. Applied to our study cohort, this index demonstrated that a high MUST score (≥1) was associated with an increased risk of late mortality after HT. These findings align with those of other studies that highlight the importance of nutritional status in cardiac patients undergoing HT. For instance, Barge-Caballero et al. [22] found that malnutrition, assessed using the Nutritional Risk Index (NRI) proposed by Buzby et al. [14], is an independent predictor of mortality and is also associated with greater susceptibility to infections and various other complications that lead to longer hospital stays. In a similar study, Krishnan et al. [6] observed that malnutrition calculated via the NRI was associated with post-transplant renal failure requiring dialysis. This finding is noteworthy, as our study cohort also showed a trend towards significance between the MUST score and the need for dialysis using CVVH within the first 24 h; further studies on a larger patient population are desirable to understand this possible correlation.

The results of our study, along with the existing literature, underscore once again how nutritional assessment and management should be an integral part of the care pathway for patients undergoing HT, even for those suffering from malnutrition (45% of patients in our cohort had a MUST score ≥ 1). Indeed, the guidelines of the International Society for Heart and Lung Transplantation [23] suggest an upper BMI limit of <30 for consideration for HT, but they do not provide specific parameters to identify the lower limit of malnutrition and determine transplant eligibility based on malnutrition. We believe that it is essential to propose nutritional strategies that can be implemented in HT centers worldwide. These strategies should aim to improve the nutritional status of patients both before and after HT. Early nutritional screening using tools like the MUST can facilitate prompt identification of at-risk patients at the time of transplant evaluation.

This approach may allow for personalized nutritional interventions, including protein supplements, vitamin and mineral supplementation, and specific dietary counseling, to improve the patient’s nutritional status. Additionally, regular monitoring of the nutritional status of patients on the transplant waiting list, with frequent updates to adapt nutritional strategies as needed, is crucial for the preoperative assessment of patients with heart insufficiency waiting for HT. After the transplant, implementing an intensive nutritional support regimen, including enteral or parenteral nutrition in cases where the patient cannot feed orally, is vital. Ensuring strict nutritional follow-up with regular visits to a clinical nutritionist to monitor and maintain adequate nutritional status can help mitigate complications. However, it should be noted that a close association between nutritional screening results and clinical outcomes does not necessarily imply that early nutritional intervention will influence them; thus, further prospective studies are encouraged to begin answering these feasibility questions.

The retrospective nature of this study represents its main limitation, as it entails potential selection bias, which may impair the accuracy of data collection. Moreover, the small sample size does not allow for a clear and definite association between malnutrition and postoperative complications, only indicating a trend of correlation and behavior. The absence of a significant correlation between MUST scores and the length of hospital stay or major postoperative complications in our patient cohort is an interesting result that warrants further investigation. This discrepancy could be due to differences in inclusion criteria, nutritional assessment methods, or surgical and postoperative management practices.

Additional studies comparing the MUST score to other nutritional tests (e.g., Short Nutritional Assessment Questionnaire, Malnutrition Screening Tool) are warranted. Furthermore, a more detailed weighted analysis including the effects of micronutrient deficiencies on early and late outcomes might aid in understanding the mechanisms underlying malnutrition in this population [24].

## 5. Conclusions

In conclusion, this study confirms that a state of malnutrition assessed by a MUST score equal to or greater than 1 before HT is not necessarily associated with increased in-hospital mortality or major postoperative complications immediately after HT; however, it is a significant factor for long-term mortality in patients undergoing HT.

## 6. Key Points

Malnutrition is a clinical condition that, as demonstrated in several studies, influences the mortality and morbidity of patients undergoing cardiac surgery, particularly those with end-stage heart failure who are undergoing HT. However, traditional malnutrition indices, such as albumin levels or BMI, are often affected by the disease itself. Therefore, it is crucial to assess the state of malnutrition in these patients using indices that are more appropriate for their condition, enabling them to benefit from pre- and postoperative treatments that optimize transplantation outcomes.

The strength of this study lies in the use of the MUST score for evaluating preoperative malnutrition status. This score is a dynamic, multi-variable, semi-quantitative tool that is useful for these patients because it is not influenced by the altered metabolic state of individuals with end-stage heart failure.

## Figures and Tables

**Table 1:** Results of univariable weighted analyses assessing the relationship between MUST scores and postoperative outcomes. Data are the OR, 95% CI, and *p*-value for categorical variables and the AME, 95% CI, and *p*-value for continuous variables. The effect was estimated per unit increase in the MUST score.

Outcome	OR/AME *	CI 95%	*p*-Value
Post-transplant ECMO first 24 h	0.92	(0.64–1.33)	0.65
Post-transplant ECMO after 24 h	0.82	(0.34–2.01)	0.67
Reintubation	1.12	(0.73–1.74)	0.60
Tracheostomy	1.16	(0.77–1.77)	0.48
Pneumonia	0.76	(0.39–1.50)	0.43
Pneumothorax or pleural effusion requiring chest drainage	1.00	(0.61–1.62)	0.99
Pericardial effusion requiring drainage	0.80	(0.53–1.20]	0.28
CVVH first 24 h	1.51	(1.01–2.25)	0.04
CVVH after 24 h	0.82	(0.54–1.25)	0.36
PEG	1.16	(0.49–2.75)	0.74
Positive blood cultures first 24 h	0.97	(0.33–2.79)	0.95
Positive blood cultures after 24 h	0.99	(0.69–1.41)	0.95
Blood cultures positive for MDR	0.98	(0.59–1.64)	0.95
Wound dehiscence	1.30	(0.69–2.44)	0.42
Cellular reject	1.20	(0.87–1.65)	0.28
Respiratory care unit rehabilitation	1.22	(0.65–2.27)	0.54
In-hospital mortality	0.95	(0.61–1.47)	0.80
Mechanical ventilation (days)	−0.5546 *	(−2.61–1.49)	0.60
ICU stay (days)	0.9432 *	(−1.68–3.57)	0.48
Hospitalization stay (days)	−1.773 *	(−7.42–3.88)	0.54

Abbreviations: n = number; ECMO = extracorporeal membrane oxygenation; CVVH = continuous venovenous hemofiltration; PEG = percutaneous endoscopic gastrostomy; MDR = multidrug resistance; ICU = intensive care unit; * AME = average marginal effect (for continuous outcomes); OR = odds ratio; CI = confidence interval.

**Table 2:** Baseline preoperative and intraoperative characteristics of enrolled patients.

Variable	*n* (%)/Median (IQR) All Patients *n* = 168
Age (years)	58.4 (49.5–65.2)
BMI (kg/mq)	24.2 (21.2–27.9)
Sex n (%)	
M	128 (76%)
F	40 (24%)
MUST score	
Low risk of malnutrition (must = 0)	92 (55%)
Moderate risk of malnutrition (must = 1)	24 (14%)
High risk of malnutrition (must ≥ 2)	52 (31%)
eGFR (mL/min)	64.3 (49-0-83.2)
Serum creatinine (mg/dL)	103.5 (87.0–127.3)
Serum albumin (g/L)	34.0 (28.0–38.8)
INR	1.7 (1.2–2.7)
Pre-transplant hospitalization	
Yes	57 (34%)
No	111 (66%)
Duration of pre-transplant hospitalization (days)	17.0 (8.5–36.5)
Pre-transplant ECMO	
Yes	17 (10%)
No	151 (90%)
Pre-transplant intracorporeal left ventricular assist device	
Yes	50 (30%)
No	118 (70%)
Pre-transplant paracorporeal left ventricular assist device	
Yes	23 (14%)
No	145 (86%)
REDO operation	
Yes	53 (32%)
No	115 (68%)
Donor age (years)	52.0 (39.8–60.3)
Ischemic time of donor organ (minutes)	220.0 (176.0–246.0)
CPB time (minutes)	198.5 (170.3–249.5)
ACC time (minutes)	80.0 (66.0–104.5)

Abbreviations: *n* = number; Q1–Q3 = 25th–75th percentile; BMI = body mass index; eGFR = estimated glomerular filtration rate; INR = international normalized ratio; ECMO = extracorporeal membrane oxygenation; MUST = Malnutrition Universal Screening Tool; CPB = cardiopulmonary bypass; ACC = aortic cross-clamp.

**Table 3:** Postoperative characteristics of patients.

Variable	*n* (%), Median (Q1–Q3) All Patients *n* = 168
Post-transplant ECMO first 24 h	
Yes	40 (24%)
No	128 (76%)
Post-transplant ECMO after 24 h	6 (4%)
Mechanical ventilation (days)	2.0 (1.0–4.0)
ICU stay (days)	5.0 (3.0–16.0)
Reintubation	
Yes	23 (14%)
No	145 (86%)
Tracheostomy	
Yes	24 (14%)
No	144 (86%)
Pneumonia	
Yes	12 (7%)
No	156 (93%)
Pneumothorax or pleural effusion requiring chest drainage	
Yes	20 (12%)
No	148 (88%)
Pericardial effusion requiring drainage	
Yes	37 (22%)
No	131 (78%)
CVVH first 24 h	
Yes	25 (15%)
No	143 (85%)
CVVH after 24 h	31 (18%)
PEG	
Yes	4 (2%)
No	164 (98%)
Positive blood cultures first 24 h	
Yes	5 (3%)
No	163 (97%)
Positive blood cultures after 24 h	42 (25%)
Blood cultures positive for MDR	
Yes	17 (10%)
No	151 (90%)
Wound dehiscence	
Yes	8 (5%)
No	160 (95%)
Cellular rejection (n patients = 158)	
0	100 (14%)
1	32 (20%)
1A	19 (12%)
1B	2 (1%)
2	2 (1%)
3A	3 (2%)
Respiratory care unit rehabilitation	
Yes	8 (5%)
No	160 (95%)
Hospitalization stay (days)	35.0 (28.0–49.3)
In-hospital mortality	
Yes	25 (15%)
No	143 (85%)

Abbreviations: *n* = number; Q1–Q3 = 25th–75th percentile; ECMO = extracorporeal membrane oxygenation; ICU = intensive care unit; CVVH = continuous venovenous hemofiltration; MDR = multidrug resistance: PEG = percutaneous endoscopic gastrostomy.

**Table 4:** Weighted Cox regression analysis to assess death at follow-up in patients with increasing MUST score.

Variable	Hazard Ratio	IC 95%	*p*-Value
Preoperative increasing MUST score	1.38	(1.04–1.83)	0.0241

Abbreviations: MUST = Malnutrition Universal Screening Tool; CI = confidence interval.

## Data Availability

The datasets presented in this article are not readily available because the data are part of an ongoing study. Requests to access the datasets should be directed to the corresponding author.
